# Gender-based vulnerability in women who inject drugs in a harm reduction setting

**DOI:** 10.1371/journal.pone.0230886

**Published:** 2020-03-30

**Authors:** Jorge Valencia, Alejandro Alvaro-Meca, Jesús Troya, Jorge Gutiérrez, Cristina Ramón, Antonio Rodríguez, Sonia Vázquez-Morón, Salvador Resino, Santiago Moreno, Pablo Ryan

**Affiliations:** 1 Harm Reduction Unit “SMASD”; Subdirección General de Adicciones; SERMAS, Madrid, Spain; 2 Non-Governmental Organization “Madrid Positivo”, Madrid, Spain; 3 Unit of Preventive Medicine and Public Health, Rey Juan Carlos University, Alcorcon, Madrid, Spain; 4 Hospital Universitario Infanta Leonor, Madrid, Spain; 5 Fundación Estatal, Salud, Infancia y Bienestar Social (FCSAI), Madrid, Spain; 6 Unit of Viral Infection and Immunity, National Center for Microbiology, Institute of Health Carlos III, Majadahonda, Madrid, Spain; 7 Department of Infectious Diseases, Ramón y Cajal Hospital, IRYCIS, University of Alcalá de Henares, Madrid, Spain; 8 Complutense de Madrid University (UCM), Madrid, Spain; 9 Instituto de Investigación Sanitaria Gregorio Marañón (IiSGM), Madrid, Spain; Medizinische Universitat Wien, AUSTRIA

## Abstract

**Background and aims:**

In comparison with men, women who use drugs (WWUD) have considerably more frequent and intense experiences with interpersonal violence, sexual abuse and trauma. The aim of this study was to identify issues related to gender-based vulnerability in a group of WWUD attended in a harm reduction facility in Madrid, Spain.

**Material and methods:**

A cross-sectional study was conducted during a screening of blood borne infections. We included WWUD (smoked or injected heroin/cocaine) who were actively screened for HIV, HBV and HCV in a harm reduction setting in Madrid (Spain) from January to December 2017. WWUD were interviewed for gender-based abuse or violence using a face-to-face questionnaire by a trained interviewer. Aspects related to their social-epidemiological condition and gender-based vulnerability were collected.

**Results:**

We included 109 women who were actively using drugs. The median age was 39 (IQR 35–47) years, 84.4% were Spanish born, 22.9% were homeless, 43 (41.7%) had ever used injected drugs, 29 (26.6%) were currently using injected drugs, and 27.1% had mental health disorders. Aspects related to gender-based vulnerability were collected. Among those surveyed, they reported having ever suffered emotional or psychological damage (88%), having experienced at least one incident of serious physical injury by a male partner (71%), and having ever suffered sexual abuse (49%). In addition, 28% had ever exchanged sex for money/drugs. When compared to women that did not use injecting drugs, those who injected drugs had more frequently exchanged sex for money/drugs (55% vs 21%, p = 0.003).

**Conclusions:**

A high proportion of WWUD suffer psychological or physical violence by partners denoting gender-based vulnerability. Interventions in harm reduction settings with a multidisciplinary and gender-based approach should be implemented.

## Background

In a recent report, evidence of intravenous drug use (IDU) was reported in 179 of 206 countries or territories. Among an estimated 15.6 million people who inject drugs (PWID), 3.2 (1.6–5.1) million are women. The percentage of women who inject drugs (WWID) varies substantially across regions, ranging from 3.1% to 33% of all PWID [1]. The prevalence of IDU among men is far higher than in women in all regions.

Evidence of risk of HIV, viral hepatitis and sexually transmitted diseases has been demonstrated in WWID with inadequate emphasis on human rights. WWID are among the most vulnerable through both unsafe injections and unprotected sex [2]. Despite the fact that current drug use is higher among males than females [3], several studies have shown that injection practices among women may put them at a higher risk [4–7]. Some reports illustrate that women who use drugs (WWUD) are sexually active and have multiple sex partners including clients of sex work. Transient relationships are reported and many of them engage in unprotected sex [8] and some type of violence [9].

WWID in the US face high levels of stigma, punishing drug laws, increasing rates of drug-related incarceration and restricted access to harm reduction services, specifically gender-sensitive services [10]. In Canada, the increase in overdoses in British Columbia has increased violence against WWID, particularly among poor, homeless, transgender and indigenous women. Rapid and severe intoxication or overdose from fentanyl-laced opioids leave them vulnerable to thefts and physical and/or sexual assault, often from the same men who offered them the drugs .0. [11,12]. Currently, there is a global need to improve data collection and reporting of gender-disaggregated information (prevalence of key infections, gender-based violence, prevention and treatment service access and program coverage) [11].

Due to the current scarcity and relevance of this information in harm reduction settings, the aim of our study was to identify issues related to gender-based vulnerability in women who used drugs and were attended in a low-threshold mobile harm reduction unit (LTMHRU).

## Material and methods

### Study sample and ethical considerations

Participants included in the study were women aged ≥18 years-old who self-reported active use of cocaine or heroin, and who were followed by a comprehensive multidisciplinary team of a LTMHRU. This unit offers several services, including needle and syringe exchange, opiate substitution therapy (OST), addiction treatment, frequent testing and treatment for infectious diseases and other comorbidities (psychiatric, chronic diseases…), prevention counselling, coverage of basic needs, and social support. All women included were recruited after their participation in a screening program for blood borne viral infections (HIV, HCV, HBV and syphilis) using dried blood spot and syphilis through of a mobile van located next to the LTMHRU in a shantytown located in Madrid (Spain), from January to December 2017. This specific group of WWUD is characterized by frequent relapses, few or shorter periods of abstinence, impaired physical conditions, poor access to standard medical care, and be socially excluded and marginalized.

### Measurements

Data on social and demographic details, history of drug abuse, sexual risk behaviors, acceptability of the screening test and a short questionnaire related to gender-based vulnerability were recorded. All WWUD who came to the screening site were proposed to participate in this study. After agreement, an informed consent was signed. Participation consisted on a confidential and voluntary interview (face-to-face) by a woman nurse hired specifically for that purpose in a closed space located inside the mobile van. The questionnaire consisted of short questions of interpersonal violence exerted by the intimate-partner or related to sexual trade (money or others benefits such as drugs, food or housing) produced over time (childhood, adult, ever during lifetime, remote, or recent) and included sexual abuse, emotional or psychological damage and physical injury. All interviews were completed in one sitting and lasted approximately 10–15 minutes and the participants were informed that they could interrupt the questionnaire at any time during the interview.

For the purpose of this study, the following definitions were considered.

- Sexual abuse during adulthood was defined as pressured or forced vaginal or anal sex using violence or being threatened or hurt with a weapon produced by any perpetrator.
- Emotional or psychological damage was defined by traumas or extraordinarily stressful events that occur repeatedly and shatter her sense of security produced by any perpetrator.
- Serious physical injury meant an injury that caused a reasonable risk of death or caused serious or permanent disfigurement or caused significant physical pain or caused serious impairment of health produced by any perpetrator.

### Ethics statements

The research project was approved by the Research Committee at Hospital Universitario Infanta Leonor (HUIL) and the Ethics Committee of Hospital General Universitario Gregorio Marañón and Instituto de Salud Carlos III (ISCIII).

No money compensation was given to patients.

### Statistical analysis

Frequencies and percentages were calculated for dichotomous variables and median and interquartile range (IQR) were calculated for continuous variables. Pearson’s chi-square tests were used to assess if issues related to gender-based vulnerability were associated with injecting drugs among WWUD. Data were collected and managed using Research Electronic Data Capture (Redcap), and analyses were performed using R software (R Foundation, Vienna, Austria).

## Results

During the period of the study, information was obtained from 529 drug users who participated in the screening program. Among them, 109 (20.6%) women who accepted to be interviewed, were included in the study. None of them refused to participate. Baseline characteristics of the women included in the study are shown in Table 1. Overall, the median age was 39 (IQR: 35–47); 92 (84.4%) were Spanish-born, 25 (22.9%) were homeless or unstably housed, and 29 (27.1%) had mental health disorders (MHD). In relation to injecting behaviors, 43 (41.7%) had ever used injected drugs and 29 (26.6%) were currently using injected drugs, 32 (29.4%) used drugs daily and 96 (88.1%) reported cocaine as the drug they had “most often used”, 24 (25.5%) had relapsed in substance use in the prior year, and 28 (27.5%) were engaged on OST.

**Table 1:** Characteristics of 109 women who use drugs*.

	N = 109
**Sociodemographic**	** **
Age, years (median, IQR)	39 (35–47)
Spanish born	92 (84.4)
Homeless**	25 (22.9)
HIV positive	7 (6.4)
Mental health disorders	29 (27.1)
**Addiction-related characteristics**	
Current way of administration of drugs	
Injected^Ϯ^	29 (26.6)
Smoked^Ϯ^	89 (81.7)
Snorted^Ϯ^	23 (21.1)
Daily use of the drugs	32 (29.4)
Intravenous drug use ever	43 (41.7)
Sharing paraphernalia**	21 (40.4)
Relapse in drugs in the last year	24 (25.5)
On OST	28 (27.5)
**Sexual behavior **	
Stable partner	47 (44.3)
High sexual risk behavior**	23 (25.6)
Use of contraceptives	59 (57.8)
Exchange sex for money/drugs	31 (28.4)

* Expressed as n (%) unless indicated otherwise

**In the past 12 months.

^Ϯ^ multiresponse, the sum exceeds 100%

IQR: interquartile range; OST: opiate substitution therapy

All WWUD interviewed reported one or more gender-based vulnerability issues. In total, 92 (88.5%) of the women interviewed had ever suffered emotional or psychological damage by a partner Seventy-four (71.2%) women reported having experienced at least one incident of serious physical injury, and 51 (49%) had ever suffered sexual abuse. Thirty-one (28.4%) women admitted to have ever exchange sex for money/drugs, though 18 (17.1%) did not answer to this question; 47 (44.3%) had a stable partner, all of whom used drugs.

The Table 2. shows the comparison of gender-based vulnerability among WWID (n = 29) and women who use non-injectable drugs (n = 80). In this analysis, no differences were found with the exception of a higher frequency of sex exchange for money/drugs in WWID (53.6%) than in those who used non-injectable drugs (20.8%) (p = 0.003).

**Table 2:** Variables associated to gender-based vulnerability among women according to the use of injectable drugs.

Variables	WWID	WWNID	p-value
	N = 29; %	N = 80; %	
Emotional or psychological damage by any partner ever	26 (96.3)	66 (85.7)	0.258
Serious physical injury by any partner ever	19 (70.4)	55 (71.4)	0.999
Sexual abuse by any partner ever	13 (48.1)	38 (49.4)	0.999
Engaged in sex exchange for money/drugs ever	15 (53.6)	16 (20.8)	0.003

WWID: women who use injectable drugs; WWNID: women who use non-injectable drugs

The Table 3. shows a comparison of variables associated to gender-based vulnerability among WWUD who live with HIV and WWUD uninfected by HIV. No differences were found among WWUD according to their HIV status.

**Table 3:** Variables associated to gender-based vulnerability among women according to their HIV status.

Variables	HIV+	HIV-	p-value
	N = 7; %	N = 102; %	
Emotional or psychological damage by any partner ever	7 (100)	85 (87.6)	0.706
Serious physical injury by any partner ever	5 (71.4)	69 (71.1)	0.999
Sexual abuse by any partner ever	5 (71.4)	46 (47.4)	0.403
Engaged in sex exchange for money/drugs ever	3 (42.9)	28 (28.6)	0.710

WWID: women who use injectable drugs; WWNID: women who use non-injectable drugs; HIV: human immunodeficiency virus

## Discussion

The prevalence of gender-based vulnerability found in our study population was extremely high and similar to that found in other studies that describe a syndemic of substance abuse, partner violence, HIV, mental illness, and social instability [13–18]. All the women who participated in this study had suffered at least one act of physical or psychological violence caused by their partners.

Overall, almost half of the WWUD in this study had a stable partner and all of them used drugs. Many cases of these relationships with other drug users act as a form of protection, although are usually abusive and economically parasitical to the women [16]. Multiple studies have found that WWID are more likely than their male counterparts to have a sexual partner who injects drugs and this may reflect the greater social isolation [19–21] and their scarce social and familiar support.

On the other hand, although we believe that the proportion of WWUD who exchanged sex for money/drugs was underestimated in our study due to the study characteristics, we found that 30% of them were engaged in transactional sex for money/drugs and it was statistically higher (53.6%) in WWID than in women who use non-injectable drugs. This subgroup of WWID faces high levels of violence, abuse and sexual assault [19, 22]; indeed, many of them sell sex to finance their own and their partner’s drug habit and often their partner exerts a significant amount of control over their sex work, decreased negotiation about condom use and increasing unsafe injection practices [2, 13].

Rates of gender-based and intimate partner violence are two to five times higher among WWID than women who do not use drugs [23]. Remarkably and similar to the findings of our study, over half of all interviewed women reported having experienced sexual violence in their lifetime [24,25] and strikingly, three-quarters of women had ever experienced some degree of physical or emotional violence by their partners. Unfortunately, this behavior is permanent because of the financial dependence on the partner for housing, drug supply, and fear [26]

These high levels of gender-based vulnerability was not significantly different for WWID and women who use non-injectable drugs. Our findings agree with those of a qualitative study that showed that sexual and reproductive health needs of women who inject drugs and those who use drugs through non-injectable routes are largely similar [8]. Indeed, gender specific needs may often be overlooked in WWUD and therefore, there is a huge unmet need to provide psychologic and social support, including financial assistance, housing assistance, self-esteem and empowerment training, sexual health education and legal advice. Currently, healthcare systems and individual providers may be poorly equipped with resources to engage abused women, especially those with active substance use disorders. It could require the development of gender-specific medical and social outreach services [13] to more effectively engage WWUD in sexual transmission and blood-borne infections and gender- based violence prevention, and insert it in women’s harm reduction settings.

Our study has several limitations to be considered. First, the sample size is small and could have limited the power to detect differences in potential covariates such as HIV status and injecting-drug use; however, the proportion of WWUD in cohort studies of PWID actively are traditionally low and women are usually under-represented. Second, this study focuses exclusively in women who were attended in a harm reduction program and with a chaotic lifestyle, which may not be generalizable to other populations of WWUD or other types of substances abuse. Third, vulnerability was not assessed using gender- violence specific scales or scores [27,28]; however, given the importance of the results and the lack of information on gender-based vulnerability related to severe substance abuse in a harm reduction setting in Madrid, Spain [15,29], we found our findings very relevant. Fourth, an information bias (interview face-to-face) is possible, but a trained female nurse and a short questionnaire could have minimized this issue. Fifth, the results on drug use and violent events were based on self-report measures rather than urine toxicology or other factual evidence. In the same way, the diagnostic of MHD was self-reported by patients and it was not assessed in-depth. This could have underestimated and explained the low prevalence found and be a bias due to this MHD is one of the most relevant vulnerability factors in WWID.

### Implications for practice and/or policy

Women who used drugs in our study suffered various forms of sexual, physical and psychological violence. We note that these results provide particularly strong evidence for the implementation of programs focused in violence support and prevention in women who use drugs in a harm reduction setting. These issues should generate policies of detection early signs of abuse or violence promoting derivation to specialized public services and strengthening women’s empowerment activities. Drug policy makers should also create specific resources including housing and phycological support for women who suffer gender-based violence and cannot or do not want to stop using drugs.

## Conclusions

In summary, a high proportion of WWUD suffer psychological or physical violence by partners denoting gender-based vulnerability that requires and urgent, multidisciplinary approach and support services adapted to the needs of these women.
